# PASSer2.0: Accurate Prediction of Protein Allosteric Sites Through Automated Machine Learning

**DOI:** 10.3389/fmolb.2022.879251

**Published:** 2022-07-11

**Authors:** Sian Xiao, Hao Tian, Peng Tao

**Affiliations:** Center for Research Computing, Center for Drug Discovery, Design and Delivery (CD4), Department of Chemistry, Southern Methodist University, Dallas, TX, United States

**Keywords:** allostery, machine learning, allosteric site prediction, automated machine learning (AutoML), deep learning

## Abstract

Allostery is a fundamental process in regulating protein activities. The discovery, design, and development of allosteric drugs demand better identification of allosteric sites. Several computational methods have been developed previously to predict allosteric sites using static pocket features and protein dynamics. Here, we define a baseline model for allosteric site prediction and present a computational model using automated machine learning. Our model, PASSer2.0, advanced the previous results and performed well across multiple indicators with 82.7% of allosteric pockets appearing among the top three positions. The trained machine learning model has been integrated with the Protein Allosteric Sites Server (PASSer) to facilitate allosteric drug discovery.

## 1 Introduction

Allostery is a fundamental process that regulates protein functional activities and is known to play a key role in biology (Gunasekaran et al. 2004). In an allosteric process, an effector molecule binds to a protein at its allosteric site, often resulting in conformational and dynamical changes (Srinivasan et al. 2014; Huang et al. 2013). Allosteric drug development is promising for many reasons: the allosteric drugs could be more selective and less toxic with fewer side effects; they can either activate or inhibit proteins; they can be used in conjunction with orthosteric drugs. Due to these advantages, the development of allosteric drugs has gradually increased in recent years (Wagner et al. 2016; Nussinov et al. 2011; Nussinov and Tsai 2013).

Several methods have been developed to detect and predict allosteric sites in proteins, such as normal mode analysis (NMA) (Panjkovich and Daura 2012), molecular dynamics (MD) simulations (Laine et al. 2010), and machine learning (ML) models (Amor et al. 2016; Bian et al. 2019; Huang et al. 2013). Several current methods are available as web servers or open-source packages, such as Allosite (Huang et al. 2013), SPACER (Goncearenco et al. 2013), PARS (Panjkovich and Daura 2014), AlloPred (Greener and Sternberg 2015), AllositePro (Song et al. 2017), and PASSer (Tian et al. 2021a). These studies have demonstrated the feasibility of allosteric site prediction models which combine pocket features and protein dynamics. As summarized by Lu et al. (2014), these studies can be classified as structure-based, dynamics-based, NMA-based, or combined prediction approaches. In structure-based approaches, such as Allosite, site descriptors describing chemical and physical properties of protein pockets are calculated as features for prediction. NMA-based approaches, such as PARS, take the ability of NMA, which can provide global modes that bear functional significance, for discovering protein sites that can mediate or propagate allosteric signals. In dynamics-based approaches, MD simulations and a two-state Ga model are used to construct a conformational or energy landscape, in which the latter can be used to calculate population distribution upon perturbation. SPACER combines dynamics-based and NMA-based approaches, which apply Monte Carlo simulations and normal mode evaluation to unravel latent allosteric sites.

The past decade has witnessed the rapid development of machine learning in chemistry and biology (Zhang et al. 2020; Chen L. et al. 2021; Tian et al. 2020; Tian et al. 2021b; Tian et al. 2022). ML methods have been shown to be superior in the classification of protein allosteric pockets. Allosite and AlloPred used support vector machine (SVM) (Suykens and Vandewalle 1999) with curated features. Chen et al. (2016) used random forest (RF) (Liaw and Wiener 2002) to construct a three-way predictive model. Our previous study (Tian et al. 2021a) used an ensemble learning method combining the results of eXtreme gradient boosting (XGBoost) (Chen and Guestrin 2016) and graph convolutional neural networks (GCNNs) (Kipf and Welling 2016).

Recently, automated machine learning (AutoML) has emerged as a novel strategy to implement machine learning methods to solve real-world problems Hutter et al. (2019). It has been widely applied in biomedical or chemistry fields like nucleic acid (Chen Z. et al. 2021), healthcare (Waring et al. 2020), and disease studies (Karaglani et al. 2020; Panagopoulou et al. 2021). As the name suggests, AutoML helps to automate the machine learning pipeline, from data processing, model selection, and ensemble to hyperparameter tuning. This saves human power from the time-consuming and iterative tasks of machine learning model development Yao et al. (2018). Also, AutoML offers the opportunities to produce simpler solutions with superior model performance (Elshawi et al. 2019).

In this study, we first defined the baseline for protein allosteric site prediction, an algorithm that identifies the pocket with the highest pocket score among all pockets detected by FPocket (Le Guilloux et al. 2009) as allosteric. This primitive baseline predictor has accuracy, precision, recall, and F1 score values of 0.968, 0.689, 0.571, and 0.624, respectively. Then, we applied two AutoML frameworks, AutoKeras (Jin et al. 2019) and AutoGluon (Erickson et al. 2020), for the prediction of protein allosteric sites. Our model is shown to be robust and powerful under various indicators with precision, recall, and F1 score values of 0.850, 0.616, and 0.701, respectively, on the test set, and 82.7% of allosteric sites in the test set are ranked among the top three positions. We also applied the well-trained model to predict allosteric sites from novel proteins that are not included in the training set and demonstrated their binding structures.

## 2 Materials and Methods

### 2.1 Protein Database

The protein data used in this work were collected from the Allosteric Database (ASD) (Huang et al. 2011). Its newest version contains a total of 1,949 entries of allosteric sites, each with different proteins and modulators Liu et al. (2020). However, data need to be filtered from ASD under certain criteria to ensure the data quality Zha et al. (2022). To ensure protein quality and diversity, Huang et al. (2013) selected 90 proteins using the previous rules: protein structures with either resolution below 3 Å or missing residues in the allosteric sites were removed, and redundant proteins that have more than 30% sequence identity were filtered out. ASBench (Huang et al. 2015), an optimized selection of ASD data, includes a core set with 235 unique allosteric sites and a core-diversity set with 147 structurally diverse allosteric sites. Here, we use 90 proteins from ASD and 138 proteins in the core-diversity set from ASBench. A total of 204 proteins were used in this study, after removing the duplicate records. The selected proteins were stored in the GitHub repository for this study.

### 2.2 Site Descriptors

FPocket, a geometry-based algorithm to identify pockets, is used to detect pockets on the surface of the selected proteins. For each of the detected pockets, 19 numerical features are calculated from FPocket (Supplementary Table S1). Compared with other web servers and open-source pocket detection packages, FPocket is superior in execution time and the ease to be integrated with other models.

For the 90 proteins from ASD, a pocket is labeled as either 1 (positive) if it contains at least one residue identified as binding to allosteric modulators or 0 (negative) if it does not contain such residues. Therefore, a protein structure may have more than one positive label. A total of 2,123 pockets were detected with 133 pockets being labeled as allosteric sites. For the 138 proteins from ASBench, a total of 3,708 pockets were detected. A pocket is labeled as 1 (positive) only if its centroid is the closest to that of the allosteric modulator, otherwise 0 (negative).

### 2.3 Automated Machine Learning

The implementation of the state-of-the-art ML methods normally requires extensive domain knowledge and experience. This process includes data preparation and preprocessing, feature engineering, model selection, and hyperparameter tuning, which are time-consuming and challenging. Automated machine learning aims to free human effort from this process.

Keras is an open-source software library that provides a Python interface for artificial neural networks. Keras offers consistent and simple APIs and provides clear and actionable error messages. It also has extensive documentation and developer guides. AutoKeras (Jin et al. 2019) is an AutoML system based on Keras, enabling Bayesian optimization to guide the network morphism for efficient neural architecture parameter search. In the current study, AutoKeras v1.0.16 is applied.

Developed by Amazon Web Services, AutoGluon (Erickson et al. 2020) automates these ML tasks and achieves the best performance. Moreover, AutoGluon includes techniques for multi-layer stacking that can further boost ML performance. AutoGluon is advantageous in: (1) simplicity: straightforward and user-friendly APIs; (2) robustness: no data manipulation or feature engineering required; (3) predictable-timing: ML models are trained within the allocated time; (4) fault-tolerance: the training process can be resumed after interruption. Also, AutoGluon is an open-source library with transparency and extensibility. Another advantage is that the AutoGluon framework uses a multi-layer stacking with k-fold bagging to reduce the model’s variance. The number of layers and the value of k are heuristically determined within the framework. AutoGluon v0.2.0 is applied in this study with 14 base models, including random forest, XGBoost, and neural network. The models are listed in Supplementary Table S2.

### 2.4 Performance Indicators

For binary classification, the results can be evaluated using a confusion matrix (Table 1).

**TABLE 1:** Binary classification results in a confusion matrix.

	Real positive	Real negative
Predicted positive	True-positive (TP)	False-positive (FP)
Predicted negative	False-negative (FN)	True-negative (TN)

Various indicators could be constructed based on the confusion matrix to quantify the model performance: (1) precision measures how well the model can predict real positive labels; (2) recall measures the ability to classify true-positive and true-negative; (3) F1 score is the weighted average of precision and recall. These indicators are calculated through Eqs 1–3. The higher the values of these indicators, the better the model’s performance.

$$\text{Precision}=\frac{\text{TP}}{\text{TP}+\text{FP}},$$ (1)

$$\text{Recall}=\frac{\text{TP}}{\text{TP}+\text{FN}},$$ (2)

$$\text{F}1\text{ score}=\frac{2\times \text{Precision}\times \text{Recall}}{\text{Precision}+\text{Recall}}.$$ (3)

## 3 Results and Discussion

### 3.1 Baseline With FPocket

FPocket detects pockets on the surface of the selected proteins and sorts them in the descending order of pocket scores, which reflect the putative capacity of the pocket to bind a small molecule. The scoring function formula in FPocket is shown in the supporting information. As described in FPocket, a training dataset containing 307 proteins was first generated to determine the weights of the five features in calculating the pocket score. These proteins are filtered based on a previous study for the evaluation of PocketFinder (An et al. 2005), which is trained on 5,616 protein–ligand complexes, including 4,711 unique proteins and 2,175 unique ligands. As proposed, PocketFinder can be used to predict ligand-binding pockets and suggest new allosteric pockets, leveraging the allosteric site prediction power to FPocket.

**FIGURE 1:**
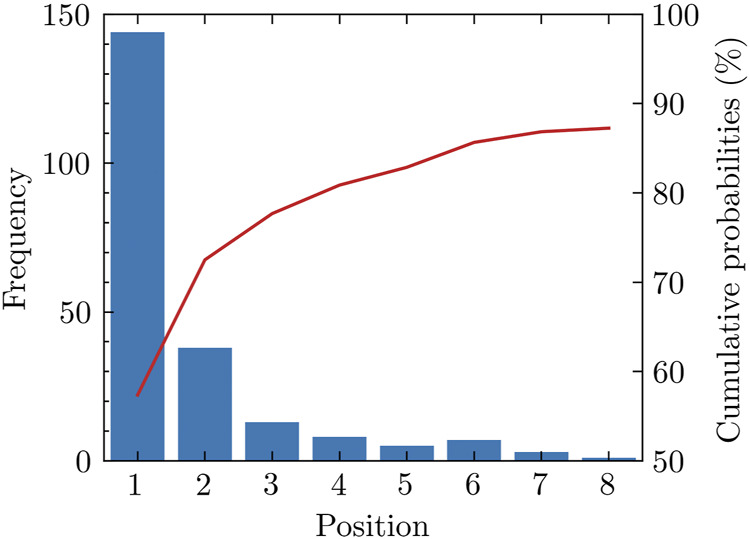
Rank of positive pockets among all pockets. Nearly 90 percent of positive pockets appear among the first eight pockets sorted by the pocket score.

We notice that many positive pockets have relatively high pocket scores. For 70.6% of the total 204 proteins used in our study, the top-ranked pocket among the pockets detected is positive in our labeling method. For 84.3% of proteins in the test set, the positive pockets are among the top three ranked positions. Among all the positive pockets, nearly 90% of them appear in the first eight positions (Figure 1).

Here, we designed a baseline for allosteric site prediction: a predictor that predicts the pocket with the highest pocket score as positive, and others as negative. We applied this baseline model to the data and evaluated the performance. The confusion matrix is shown in Table 2. The accuracy, precision, recall, and F1 score values are 0.968, 0.706, 0.574, and 0.633, respectively.

**TABLE 2:** Confusion matrix of the baseline predictor.

	Real positive	Real negative
Predicted positive	144	60
Predicted negative	107	4844

A model could be evaluated as useful if it either has higher performance indicator values (classifying power) or higher top three probabilities (ranking power) than this baseline predictor model.

### 3.2 Model Selection and Fine-Tuning on the Validation Set

**FIGURE 2:**
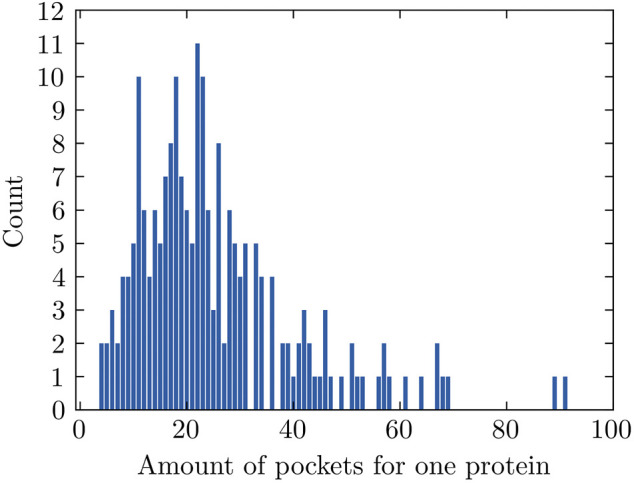
Amounts of pockets for proteins. The amount varies from 4 to 91.

The number of pockets that FPocket detects for each individual protein ranges between 4 and 91 for 204 proteins used in this study and has an average value of 25 (Figure 2). The pockets with positive labels only account for 4.87% (251 out of 5,155) in all pockets, making this dataset highly imbalanced. Data imbalance happens in a classification problem where the samples are not equally distributed among classes. This could lead to unsatisfactory model performance because the trained machine learning model might not learn sufficiently from the limited minority examples.

There are mainly two effective ways, over-sampling and under-sampling, to handle an imbalanced dataset (Lemaître et al. 2017). Over-sampling expands the size of the minority class by randomly duplicating existing examples or generating new but similar examples. However, this could result in overfitting for some machine learning models. Also, in the context of protein allosteric sites, the generated allosteric sites may not be biologically reasonable. Due to these reasons, under-sampling was applied to adjust the composition of the training data in the following procedure.

**FIGURE 3:**
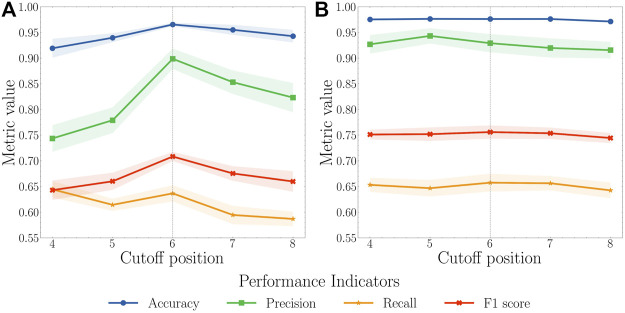
**(A)** AutoKeras and **(B)** AutoGluon models performance for all pockets of the proteins in the validation set based on different cutoff positions. The cutoff value for the training set ranges from 4 to 8. Each model was trained in 10 independent runs for each value. The mean and standard deviation of each metric were calculated. A cutoff of 6 was considered reaching a balance between recall and precision with the highest F1 score.

We first randomly split the selected 204 proteins into a training set with 122 proteins, a validation set with 41 proteins, and a test set with 41 proteins. To balance the training process, we only kept a certain number, referred to as the cutoff position, of top pockets based on their pocket scores generated by FPocket for each protein in the training set. For example, if the cutoff position is set to 5, only the first five pockets sorted by FPocket for proteins in the training set were used for the model training purpose. For cutoff positions from 4 to 8, both AutoKeras and AutoGluon models are trained and validated (Figure 3). The pocket descriptors generated by FPocket were used as features. Whether a pocket is allosteric or not according to ASD is represented as 1 for allosteric or 0 for nonallosteric. In the validation and test sets, a predicted value above 0.5 indicates an allosteric site, and a predicted value below 0.5 indicates a nonallosteric site.

Based on AutoKeras and AutoGluon model performance using cutoff values ranging 4–8, the value of 6 leads to the balance between the precision and recall with the highest F1 score. When the cutoff is smaller than 6, the unsatisfactory performance might result from insufficient data for models to learn. When the cutoff is larger than 8, the performance starts to drop because of the unbalanced and low-quality data. Therefore, the cutoff value of 6 was selected to produce the final model.

In the final model, the mean values of accuracy, precision, recall, and F1 score for the AutoKeras model were calculated as 0.955, 0.853, 0.595, and 0.675, respectively. These values for the AutoGluon model are 0.976, 0.919, 0.656, and 0.754, respectively. The results show that the AutoGluon model has a better performance than the AutoKeras model and thus was selected for further test and final deployment.

### 3.3 Test Set Performance

The final AutoGluon model using the cutoff position as 6 was tested on the test set, where the model was used to evaluate all the detected pockets. The metric values shown are comparable to its performance using the validation set (Table 3), indicating the good prediction power of this model.

**TABLE 3:** Classifying power and ranking power of AutoGluon models on the test sets.

Indicator	Mean value	Top position	Mean value
Precision	0.850	Top 1	65.1%
Recall	0.616	Top 2	77.8%
F1 score	0.701	Top 3	82.7%

It is also expected that a powerful machine learning model is capable of ranking allosteric sites in the top positions. In the current study, we evaluated the ranking power of our models by calculating the ranking probabilities of the allosteric sites at the top 1, 2, and 3 positions. The probabilities of allosteric sites, shown in Table 3, indicate that the final prediction model could rank the known allosteric sites among the top three positions for the majority of the test set. Taking the classifying power and ranking power together, our method has a great performance on allosteric site predictions.

### 3.4 Novel Protein Prediction

**FIGURE 4:**
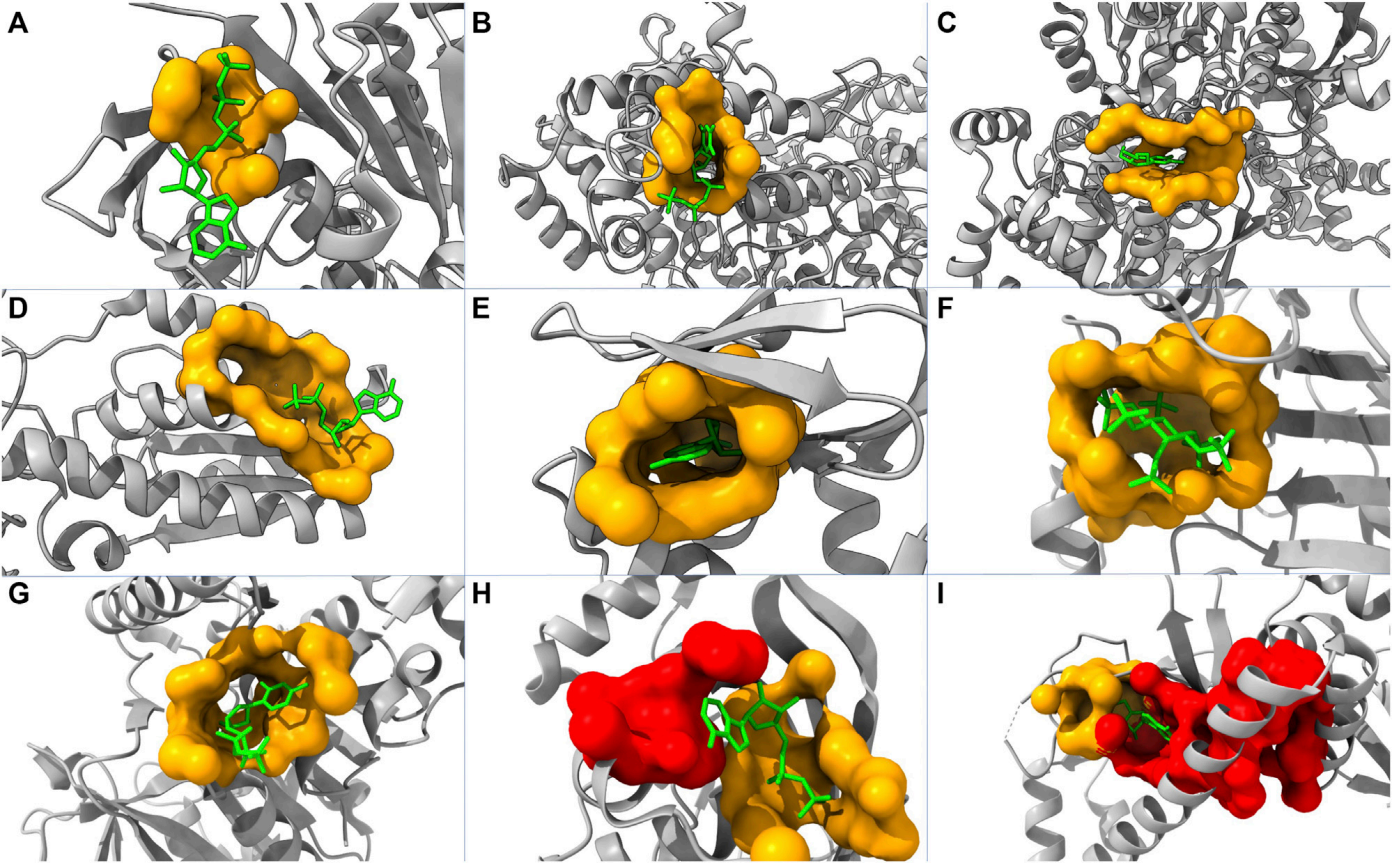
Structures of nine proteins with modulators and predicted pockets. PDB IDs of these proteins are: **(A)** 2FPL, **(B)** 2R1R, **(C)** 3BCR, **(D)** 4PFK, **(E)** 1Q5O, **(F)** 3PEE, **(G)** 4HO6, **(H)** 1XMV, and **(I)** 2OZ6. The yellow pockets are labeled as allosteric, and the lime molecules are modulators. For **(A–G)**, the allosteric pockets are successfully predicted as top one by our model. For **(H,I)**, the red pockets are predicted as the first place, and the allosteric pockets are predicted as the second place.

To further evaluate the performance of our model, we tested our model using 50 randomly picked proteins that are in the core set but not included in the core-diversity set in ASBench. Among these proteins, 22, 11, and 3 of their allosteric sites are ranked as first, second, and third, respectively. This leads to 72% of the additional test set with their true allosteric sites being ranked among the top three by our model. We also plot nine structures highlighting predicted allosteric sites and modulators (Figure 4). Our model successfully predicted allosteric sites as the top site for seven out of these proteins (Figures 4A–G), with the probabilities of 58.94%, 79.40%, 78.30%, 82.16%, 95.78%, 96.12%, and 85.11%, respectively. For protein in Figure 4H, the top pocket has a probability of 80.37%, and the real allosteric site is predicted at the second place with a probability of 77.20%. For protein in Figure 4I, the top pocket has a probability of 77.24%, and the real allosteric site is predicted at the second place with a probability of 51.09%.

In some cases, the fallaciously predicted top one pockets are close to and even merge into the pocket labeled as allosteric (Figures 4H, I). Consequently, it is not straightforward to determine whether the predicted top one pockets are false-positive. This complication of model interpretation could result from the data preprocessing (pocket detection and pocket labeling). In reality, two pockets might collectively act as one allosteric site in a biological process but being identified as two individual pockets in our model.

### 3.5 Web Server

The model has been integrated into the Protein Allosteric Site Server. The server can be either accessed at https://passer.smu.edu or through the command line. Here is an example using the command line to test the chain A of protein 5DKK using the AutoML model.

# !/bin/bash

curl −X POST \

−d pdb=5dkk −d chain=A −d model=autoML \

https://passer.smu.edu/api

**FIGURE 5:**
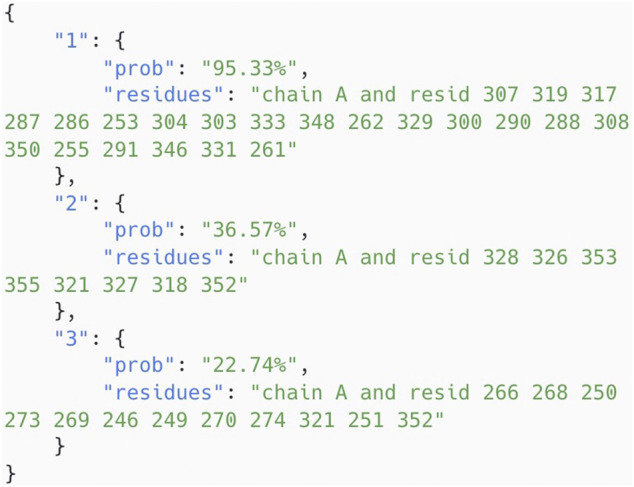
Allosteric probability results of chain A of protein 5DKK returned by command line API of PASSer.

This returns the top 3 pocket probabilities with residues in the json format, as shown in Figure 5, which can be easily parsed for further usage. Therefore, this provides a chance for large-scale searching applications for allosteric drug discovery.

## 4 Conclusion

Several machine learning-based methods have been developed for allosteric site prediction over the past few years. In this study, we applied an emerging ML technique, automated machine learning, to further improve the performance of protein allosteric site prediction models. The AutoML framework is capable of automating the machine learning model pipeline. The developed allosteric site prediction model, PASSer2.0, performs well under multiple indicators and is shown to have a good ranking power with a high percentage of ranking allosteric sites at top positions.

## Data Availability

The datasets presented in this study can be found in online repositories. The names of the repository/repositories and accession number(s) can be found in the article/Supplementary Material.
