# Supplementary material for: PASSer2.0: Accurate Prediction of Protein Allosteric Sites Through Automated Machine Learning
Source: Front Mol Biosci. 2022 Jul 11;9:879251. doi: 10.3389/fmolb.2022.879251 (PMC9309527; doi:10.3389/fmolb.2022.879251)
Supplement: Supplementary file 1 [file DataSheet1.PDF]

Supporting Information for PASSer2.0: Accurate Prediction of Protein Allosteric Sites Through Automated Machine Learning

1 SUPPLEMENTARY TABLES

Table S1. Features generated by FPocket algorithm

No.	Feature
1	Score
2	Druggability Score
3	Number of Alpha Spheres
4	Total SASA
5	Polar SASA
6	Apolar SASA
7	Volume
8	Mean local hydrophobic density
9	Mean alpha sphere radius
10	Mean alp. sph. solvent access
11	Apolar alpha sphere proportion
12	Hydrophobicity score
13	Volume score
14	Polarity score
15	Charge score
16	Proportion of polar atoms
17	Alpha sphere density
18	Cent. of mass - Alpha Sphere max dist
19	Flexibility

Table S2. Machine learning models used in the AutoGluon framework.

No.	Model
1	KNeighborsDist_BAG_L1
2	KNeighborsUnif_BAG_L1
3	ExtraTreesGini_BAG_L1
4	CatBoost_BAG_L1
5	LightGBMXT_BAG_L1
6	NeuralNetMXNet_BAG_L1
7	WeightedEnsemble_L2
8	ExtraTreesEntr_BAG_L1
9	RandomForestEntr_BAG_L1
10	NeuralNetFastAI_BAG_L1
11	LightGBM_BAG_L1
12	RandomForestGini_BAG_L1
13	LightGBMLarge_BAG_L1
14	XGBoost_BAG_L1

2 SUPPLEMENTARY EQUATIONS

The scoring function for pocket score calculation is shown below. Other alternatives are also shown in https://github.com/Discngine/fpocket/blob/master/src/pscoring.c#L241-L249.

```
score =
    - 0.03783394
    + 0.48461469 * (float) pdesc->nas_norm
    + 0.09093926 * (float) pdesc->as_density
    + 0.0004155899 * (float) pdesc->convex_hull_volume
    - 0.003995233 * (float) pdesc->surf_pol_vdw14
    - 0.004072336 * (float) pdesc->surf_apol_vdw14;
```
